# Diagnostic and Invasive Colonoscopies Do Not Increase the Risk of Prosthetic Joint Infection After Reverse Shoulder Arthroplasty

**DOI:** 10.7759/cureus.80491

**Published:** 2025-03-12

**Authors:** Matthew T Eisenberg, Clayton Hui, Colby Nielsen, Anup Shah, Evan S Lederman

**Affiliations:** 1 Orthopedic Surgery, University of Arizona College of Medicine - Phoenix, Phoenix, USA; 2 Orthopedics, University of Arizona College of Medicine - Phoenix, Phoenix, USA; 3 Orthopaedic Surgery, University of Arizona College of Medicine - Phoenix, Phoenix, USA

**Keywords:** colonoscopy, health insurance claims database, prosthetic joint infection (pji), reverse total shoulder arthroplasty, revision

## Abstract

Introduction

Reverse shoulder arthroplasty (RSA) is increasingly used to manage various degenerative and traumatic shoulder conditions. Prosthetic joint infection (PJI) remains a rare but serious complication, occurring in approximately 1-4% of cases. Given that colonoscopy can cause transient bacteremia - a potential risk factor for PJI - the current study aimed to determine whether undergoing diagnostic or invasive colonoscopy within one year after RSA is associated with an increased risk of PJI or all-cause revision surgery.

Methods

A retrospective cohort study was conducted using the PearlDiver All Payer Claims Database (MARINER). Patients who underwent RSA with at least three years of follow-up were identified and stratified into three groups: a control group (no colonoscopy), a diagnostic colonoscopy group, and an invasive colonoscopy group. The colonoscopy occurred within one year after undergoing the index procedure. Demographics, including age and sex, along with comorbidity data (using the Elixhauser Comorbidity Index), were collected. The primary outcomes assessed were the incidence of PJI and the rate of all-cause revision at 3 years postoperatively.

Results

A total of 1,244 patients in the diagnostic colonoscopy group, 2,973 in the invasive colonoscopy group, and 74,309 in the control group were identified. At three years postoperatively, the incidence of PJI was not significantly different between the diagnostic (3.94% vs. 3.29%, p = 0.20) or invasive (3.8% vs. 3.29%, p = 0.13) groups compared to controls. However, while the rate of all-cause revision in the diagnostic group (7.32% vs. 7.53%, p = 0.78) did not differ significantly from controls, the invasive colonoscopy group approached statistical significance (8.48% vs. 7.53%, p = 0.05) in univariate analysis and was statistically significant in multivariate analysis (OR = 1.63, p ≤ 0.01).

Conclusion

This study found that undergoing a diagnostic or invasive colonoscopy within one year after RSA does not increase the risk of PJI at three years postoperatively. However, patients who underwent invasive colonoscopy exhibited a higher rate of all-cause revision, which was statistically significant in multivariate analysis. These findings suggest that routine colonoscopy screening should not be deferred in RSA patients due to infection concerns, but the increased risk of revision following invasive colonoscopy highlights the need for further research to determine potential underlying factors.

## Introduction

Shoulder arthroplasty is a successful management option utilized for the treatment of several different degenerative and traumatic pathologies. It is increasingly performed in the United States [1-3]. Between 2017 and 2025, the number of shoulder arthroplasties performed is projected to increase by approximately 235% [3]. This increase is in part due to the increasing utilization of reverse total shoulder arthroplasty (RSA) [3]. Previous research utilizing the Shoulder Arthroplasty Registry [4] reported the median age of those undergoing shoulder arthroplasty to be 70 years old [5]. Specifically, in the RSA population, the mean age has been increasing, with the largest increase in utilization seen in patients >70 years old [6].

Prosthetic joint infection (PJI) after shoulder arthroplasty is relatively rare, with an incidence reported between 1% and 4%, but it can be a devastating complication leading to significant morbidity [7-9]. Colonoscopy has been shown to be associated with a risk of transient bacteremia, which is an established risk factor for developing PJI [10]. In the total joint arthroplasty literature, there has been evidence that shows that gastrointestinal endoscopy may lead to an increased risk of PJI [11]. However, research has been conflicting [12]. The United States Preventive Services Task Force (USPTF) currently recommends colonoscopy screening for those aged 45-75 years old [13].

The purpose of the current study was to determine if diagnostic or invasive colonoscopy was a risk factor for the diagnosis of PJI or the increased rate of all-cause revision in the setting of RSA. We hypothesized that those undergoing invasive colonoscopy would have an increased rate in the diagnosis of PJI and all-cause revision.

## Materials and methods

A retrospective cohort of RSA patients was gathered using the PearlDiver All Payer Claims Database (MARINER) (PearlDiver Technologies, Colorado Springs, Colorado, USA). The database includes 161 million patient records from June 2010 to April 30, 2022. PearlDiver has unique patient identifier codes allowing for longitudinal follow-up and includes claims billed to all payer types. Current Procedural Terminology (CPT®), ICD-9/10 diagnosis, and ICD-9/10 procedure codes were used to identify the study cohort, comorbidities, and outcomes (Appendix 1-3). Inclusion criteria for the study were patients who underwent RSA, the index procedure, with at least three years of follow-up. The cohort was then stratified by colonoscopy type (control vs. diagnostic vs. invasive). The control cohort did not undergo a colonoscopy. The diagnostic colonoscopy cohort had no disruption of the colonic mucosa during the colonoscopy. The invasive colonoscopy cohort consists of patients who had a resection, biopsy, or removal of a tumor, polyp, or lesion. The colonoscopy occurred within one year after undergoing the index procedure. PearlDiver is a de-identified database, and the study was exempted from Institutional Review Board approval.

Age and sex, alongside the Elixhauser Comorbidity Index (ECI), were utilized to compare the cohorts and their comorbidity burden. The ECI includes congestive heart failure, arrhythmias, valvular disease, pulmonary circulatory disorders, peripheral vascular disease, hypertension, paralysis, other neuro disorders, chronic obstructive pulmonary disease, diabetes, hypothyroidism, chronic kidney disease, liver disease, peptic ulcer disease, lymphoma, metastatic cancer, nonmetastatic cancer, rheumatoid arthritis and cardiovascular disease, coagulopathy, fluid and electrolyte disorders, anemia due to blood loss, anemia due to deficiency, alcohol abuse, drug abuse, psychoses, depression, obesity, and smoking status. The primary outcomes of this study were all-cause revision and cumulative PJI incidence rates at three years after the index procedure.

Statistical analysis

R statistical software was used to perform univariate and multivariate analyses (versuiin 4.2.3, R Core Team, 2023). Univariate analysis for categorical and continuous variables were performed using chi-squared tests and Student’s t-tests, respectively. A p-value of less than 0.05 was considered significant. ​To account for potential confounding variables, we employed multivariate logistic regression. This analysis included patient demographics and comorbidities as independent variables and was performed on outcomes that had shown a p-value of less than 0.10 in univariate analyses. Odds ratios (OR) and confidence intervals (CI) were reported for the multivariate analysis, with a p-value of less than 0.05 being significant.

## Results

A total of 1,244 individuals were identified who underwent a diagnostic colonoscopy within one year after RSA. A total of 2,973 individuals were identified who underwent invasive colonoscopy within one year after RSA. These cohorts were compared to a cohort of 74,309 patients who underwent RSA and did not undergo colonoscopy. The demographic and comorbidity data of each cohort can be seen in Table 1.

**Table 1:** Demographics and comorbidities of patients who underwent diagnostic or invasive colonoscopy within one year after reverse shoulder arthroplasty. A Student's t-test was utilized to compare the average Elixhauser Comorbidity Index score between the diagnostic and invasive colonoscopy cohorts and the control cohort. A p-value of 0.05 was considered significant. CHF: congestive heart failure, COPD: chronic obstructive pulmonary disease, ECI: Elixhauser Comorbidity Index

Patient demographics	Diagnostic colonoscopy			Invasive colonoscopy			No colonoscopy	
	n	%	p-value	n	%	p-value	n	%
Total	1244			2973			74309	
Median age range (years)	69.6			70.0			68.9	
Female	805	64.71		1712	57.58		47311	63.67
Male	439	35.29		1261	42.42		26998	36.33
Elixhauser Comorbidity Index								
CHF	152	12.22		369	12.41		7407	9.97
Cardiac arrhythmias	311	25.00		817	27.48		17798	23.95
Valvular disease	287	23.07		727	24.45		15132	20.36
Hypertension	1085	87.22		2639	88.77		63810	85.87
Other neurological disorders	92	7.40		238	8.01		5472	2.06
Paralysis	31	2.49		69	2.32		1532	34.71
COPD	498	40.03		1244	41.84		25792	34.71
Diabetes	572	45.98		1422	47.83		31103	41.86
Hypothyroidism	388	31.19		910	30.61		21960	29.55
Chronic kidney disease	235	18.89		532	17.89		12468	16.78
Liver disease	183	14.71		455	15.30		8449	11.37
Peptic ulcer disease	103	8.28		222	7.47		3940	5.30
Lymphoma	29	-		56	1.88		969	1.30
Metastatic cancer	31	2.49		74	2.49		1224	1.65
Cancer	209	16.80		539	18.13		10682	14.38
Rheumatoid arthritis	110	8.84		250	8.41		6125	8.24
Fluid and electrolyte disorders	423	34.00		965	32.46		23118	31.11
Blood loss anemia	74	5.95		132	4.44		2831	3.81
Deficiency anemia	260	20.90		542	18.23		10782	14.51
Drug abuse	112	9.00		219	7.37		4652	6.26
Psychoses	59	4.74		98	3.30		2331	3.14
Depression	532	42.77		1245	41.88		27404	36.88
Obesity	470	37.78		1269	42.68		26225	35.29
Tobacco use	471	37.86		1326	44.60		28117	37.84
Average ECI score	6.52		<0.001	6.55		<0.001	5.7	
Standard deviation for the ECI score	3.87			3.79			3.45	

At three years postoperatively, the diagnosis of PJI was not significantly different in either the diagnostic (3.94% vs. 3.29%, p = 0.20) or invasive (3.8% vs. 3.29%, p = 0.13) colonoscopy cohort as compared to the control cohort who did not undergo colonoscopy. The rate of all-cause revision at three years postoperatively was not significantly different between the diagnostic colonoscopy cohort and the control cohort (7.32% vs. 7.53%, p = 0.78). However, in the invasive colonoscopy cohort, the revision rate was higher and approached significance as compared to the control group (8.48% vs. 7.53%, p = 0.05) (Table 2). 

**Table 2:** Univariate analysis of the diagnostic and invasive colonoscopy cohorts as compared to the control cohort in terms of the revision and diagnosis of prosthetic joint infection at three years postoperatively. A Student's t-test was utilized to compare the average incidence of revision and prosthetic joint infection (PJI) at three years postoperatively between the diagnostic and invasive colonoscopy cohorts to the control cohort. A p-value of 0.05 was considered significant.

	Diagnostic colonoscopy			Invasive colonoscopy			No colonoscopy	
	n	%	p-value	n	%	p-value	n	%
Total	1244			2973			74309	
Three-year revision	91	7.32	0.78	252	8.48	0.05	5595	7.53
Three-year PJI	49	3.94	0.20	113	3.80	0.13	2444	3.29

Multivariate analysis revealed significance in comparing all-cause revision in the invasive colonoscopy cohort compared to the control cohort at three years postoperatively (OR = 1.63, p ≤ 0.01).

## Discussion

This study demonstrated that at three years postoperatively, the rate of PJI was not significantly different in either the diagnostic (3.94% vs. 3.29%, p = 0.20) or invasive (3.8% vs. 3.29%, p = 0.13) colonoscopy cohort when compared to the control cohort. Similarly, the rate of all-cause revision was not significantly different between the diagnostic colonoscopy cohort and the control cohort (7.32% vs. 7.53%, p = 0.78). However, patients in the invasive colonoscopy cohort had a higher rate of revision, which approached statistical significance when compared to the control group (8.48% vs. 7.53%, p = 0.05). Furthermore, multivariate analysis confirmed a significant association between invasive colonoscopy and all-cause revision (OR = 1.63, p < 0.01). The results of this study can help physicians with counseling patients and encouraging them to not defer their regular screening colonoscopy in the setting of RSA given the lack of increased risk of PJI. However, further research is needed to determine why there was an increased risk of all-cause revision in the invasive colonoscopy group.

PJI is a challenging complication of any joint arthroplasty given the significant morbidity it carries for patients. Mitigating the risk of PJI is thus crucial for arthroplasty surgeons. Colonoscopy has been identified as a risk for PJI in the setting of total joint arthroplasty, given the transient bacteremia that has been documented following this procedure [10,14,15]. Direct correlation between colonoscopy and PJI has only been assessed in a limited fashion. Forlenza et al. reported that invasive endoscopy within two months of TKA and one month of THA was associated with significantly increased odds of PJI, using the PearlDiver database [11]. Chiu et al., however, also using the PearlDiver database, found that after UKA, TKA, and THA, there was no increased risk of septic revision following diagnostic colonoscopy and that invasive colonoscopy was, surprisingly, protective of revision [12]. Our findings in the upper extremity are opposite of this report as we observed an increase in all-cause revision of RSA in the invasive colonoscopy group in the multivariate analysis. Both our control group revision rate and our invasive colonoscopy group revision rate of 7.53% and 8.48%, respectively, were comparable to reported revision rates of 8-12% [16-18]. In addition, the rate of PJI in our study is comparable to the generally reported 3-4% [9].

There does not appear to be any physiological connection between invasive colonoscopy and revision RSA that would suggest why we observed an increase in all-cause revision rates after invasive colonoscopy but no increase in PJI. We did control for patient comorbidities in the various groups and found no differences, indicating there was no difference in the apparent health status of individuals in these groups. The ECI could have failed to account for all possible comorbidities, which may have increased the risk for revision.

This study is not without limitations. As this study utilized administrative claims data to determine the risk of PJI and revision after shoulder arthroplasty, medical records were not able to be reviewed. This study found that invasive colonoscopy performed within one year after obtaining an RSA increased the risk of all-cause revision at three years. There is no obvious biological mechanism to explain this finding, as we also found that there was no increased risk in PJI. As this is a database study, there is the possibility that human error in miscoding could have altered our results.

## Conclusions

This study found that undergoing a diagnostic or invasive colonoscopy within one year after RSA does not increase the risk of PJI at three years postoperatively. However, patients who underwent invasive colonoscopy exhibited a higher rate of all-cause revision, which was statistically significant in multivariate analysis. These findings suggest that routine colonoscopy screening should not be deferred in RSA patients due to infection concerns. However, the increased risk of revision following invasive colonoscopy highlights the need for further research to determine potential underlying factors.

## Appendices

Current Procedural Terminology (CPT®) procedure codes

**Table 3:** Colonoscopy codes

Diagnostic colonoscopy	Colonoscopy with intervention
CPT-45378	CPT-45380
	CPT-45384
	CPT-45385
	CPT-45390

ICD-9/10 codes

**Table 4:** Revision codes

Code	Description
ICD-9-P-8001	Arthrotomy for the removal of prosthesis without replacement, shoulder
ICD-9-P-8011	Other arthrotomy – shoulder
0RWK08Z	Revision of spacer in the left shoulder joint, open approach
0RWK0JZ	Revision of synthetic substitute in the left shoulder joint, open approach
0RWK3JZ	Revision of synthetic substitute in the left shoulder joint, percutaneous approach
0RWK4JZ	Revision of synthetic substitute in left shoulder joint, percutaneous endoscopic approach
0RWKXJZ	Revision of synthetic substitute in left shoulder joint, external approach
0RRK00Z	Replacement of left shoulder joint with reverse ball and socket synthetic substitute, open approach
0RRJ00Z	Replacement of right shoulder joint with reverse ball and socket synthetic substitute, open approach
0RWJ08Z	Revision of spacer in right shoulder joint, open approach
0RWJ0JZ	Revision of synthetic substitute in right shoulder joint, open approach
0RWJ3JZ	Revision of synthetic substitute in right shoulder joint, percutaneous approach
0RWJ4JZ	Revision of synthetic substitute in right shoulder joint, percutaneous endoscopic approach
0RWJXJZ	Revision of synthetic substitute in right shoulder joint, external approach
0RRKOJZ	Replacement of left shoulder joint with synthetic substitute, open approach
0RRJ0JZ	Replacement of right shoulder joint with synthetic substitute, open approach
0RHK08Z	Insertion of spacer into left shoulder joint, open approach
0RHK38Z	Insertion of spacer into left shoulder joint, percutaneous approach
0RHK48Z	Insertion of spacer into left shoulder joint, percutaneous endoscopic approach
0RPK08Z	Removal of spacer from left shoulder joint, open approach
0RPK38Z	Removal of spacer from left shoulder joint, percutaneous approach
0RPK3JZ	Removal of synthetic substitute from left shoulder joint, percutaneous approach
0RPK0JZ	Removal of synthetic substitute from left shoulder joint, open approach
0RPK4JZ	Removal of synthetic substitute from left shoulder joint, percutaneous endoscopic approach
0RHJ08Z	Insertion of spacer into right shoulder joint, open approach
0RHJ38Z	Insertion of spacer into right shoulder joint, percutaneous approach
0RHJ48Z	Insertion of spacer into right shoulder joint, percutaneous endoscopic approach
0RPJ08Z	Removal of spacer from right shoulder joint, open approach
0RPJ38Z	Removal of spacer from right shoulder joint, percutaneous approach
0RPJ0JZ	Removal of synthetic substitute from right shoulder joint, open approach
0RPJ3JZ	Removal of synthetic substitute from right shoulder joint, percutaneous approach
0RPJ4JZ	Removal of synthetic substitute from right shoulder joint, percutaneous endoscopic approach

ICD-9/10 codes  

**Table 5:** Prosthetic joint infection codes

Code	Description
ICD-9-D-99666	Infection and inflammatory reaction due to internal joint prosthesis
ICD-9-D-99667	Infection and inflammatory reaction due to other internal orthopedic device, implant, and graft
ICD-9-D-99660	Infection and inflammatory reaction due to unspecified device, implant, and graft
ICD-9-D-99669	Infection and inflammatory reaction due to other internal prosthetic device, implant, and graft
ICD-9-D-73011	Chronic osteomyelitis
ICD-9-D-73021	Unspecified osteomyelitis
ICD-9-D-73091	Unspecified infection of bone
ICD-9-D-73081	Other infections involving bone in diseases classified elsewhere, shoulder region
ICD-9-D-99851	Infected postoperative seroma
ICD-10-D-T8579XA	Infection and inflammatory reaction due to other internal prosthetic devices, implants and grafts, initial encounter
ICD-10-D-T8450XA	Infection and inflammatory reaction due to unspecified internal joint prosthesis, initial encounter
ICD-10-D-T8460XA	Infection and inflammatory reaction due to internal fixation device of unspecified site
ICD-10-D-T847XXA	Infection and inflammatory reaction due to other internal orthopedic prosthetic devices, implants and grafts, initial encounter
ICD-10-D-T8579XA	Infection and inflammatory reaction due to other internal prosthetic devices, implants and grafts, initial encounter (duplicate)
ICD-10-D-T81.4XXA	Infection following a procedure, initial encounter
ICD-10-D-T8459XA	Infection and inflammatory reaction due to other internal joint prosthesis, initial encounter
ICD-10-D-M60.011	Infective myositis, right shoulder
ICD-10-D-M60.012	Infective myositis, left shoulder
ICD-10-D-M60.019	Infective myositis, unspecified shoulder
ICD-10-D-M86.119	Other acute osteomyelitis, unspecified shoulder
ICD-10-D-M86.219	Subacute osteomyelitis, unspecified shoulder
ICD-10-D-M86.619	Other chronic osteomyelitis, unspecified shoulder
